# Mild traumatic brain injury and delayed alteration of memory processing

**DOI:** 10.3389/fnins.2015.00369

**Published:** 2015-10-14

**Authors:** Philip P. Foster

**Affiliations:** ^1^Department of Nanomedicine and Biomedical Engineering, The Brown Foundation, Institute of Molecular Medicine for the Prevention of Human Diseases, The University of Texas Health Science Center at Houston - Medical SchoolHouston, TX, USA; ^2^Pulmonary, Sleep and Critical Care Medicine, Department of Internal Medicine, The University of Texas Health Science Center at Houston - Medical SchoolHouston, TX, USA

**Keywords:** MTBI, hippocampus, relational memory, fMRI, default mode network, salience network, single nucleotide polymorphism, BDNF

A brain traumatism may result from focal impact upon the head and/or sudden acceleration/deceleration kinetic forces applied to the brain within the rigid skull, or by a complex association of both. Approximately 500,000 to 3 million US cases occur per year. A major problem is that traumatic brain injury (TBI) classified as “mild” may not be reflected by lesions on conventional neuroimaging scans which appear “normal” albeit some mild TBI patients with “normal scans” may express long-term cognitive deficits (Irimia et al., 2012b). This raises overarching questions: would mild TBI result in cognitive deficits years later? How would memory consolidation and long-term potentiation then be affected? Monti et al. attempt to answer some of these questions in the article which appeared in *Vol. 5* of *Frontiers in Aging Neuroscience* (Monti et al., 2013) by studying the relational memory, i.e., the performance of acquiring and retaining memory to construct association of elements of a scene or events in patients having experienced TBI early in their lives. Relational memory impairment is one of the behavioral phenotypes of mild TBI following the selective damage of specific intrinsic connectivity networks, ICNs (Barbey et al., 2015). The interest of Monti's study is the determination of post-TBI disrupted networks by visualization of their connectivity which will ultimately serve as patient-personalized diagnostic tools (Irimia et al., 2012a).

The salience (SN) and the default mode networks (DMN) underlie some mechanisms of memory performance tested in their article using encoding trials via the presentation of visual scenes to memorize (Monti et al., 2013) and entails the appropriate functioning of those networks. An injury to a single white matter tract can lead to an alteration of the encoding and recognition processes. A key player in memory processing is the hippocampus which is also the center of white matter efferent or afferent projections. In humans, neurons in the hippocampus are more sensitive to post-traumatic degeneration and prone to apoptotic decay (Beauchamp et al., 2011). Even after a single TBI event, hippocampal atrophy may arise within months (Fotuhi et al., 2012). The remarkable cross-sectional study by Monti et al. was designed to correlate the history of mild TBI early in life to the relational memory networks dysfunction unveiled later in midlife. Careful subjects' inclusion/exclusion criteria enable separation of the cumulative effect of aging and mild TBI. They report a reduction of: (1) Bilateral hippocampal volume; and (2) Neural activity for the retrieval process of successful memory recognition. The fMRI data showed a decreased BOLD response in several areas of the brain such as the pre-frontal cortex (PFC), cingulate cortex and precuneus. The dysfunction of large-scale neural networks connecting PFC and atrophic hippocampus may indeed lead to memory deficits. Furthermore, Monti et al. (2013) noted a reduction of BOLD response in mild TBI patients compared with the control group in specific areas of DMN. Noticeably, those DMN areas are also the site of amyloid-β plaques depositions during normal aging.

A reasonable conjecture is that DAI may prompt the disconnection of specific ICNs (Barbey et al., 2015) as evidenced by tensor diffusion imaging enabling mapping of the mild TBI-induced disruption of long-distance white matter tracts and structural damage (Mayer et al., 2010; Bartnik-Olson et al., 2014; Zhu et al., 2014), and further neurological impairment (Sharp et al., 2014). Intrinsic connectivity network anomalies have also been observed in resting-state functional magnetic resonance imaging (fMRI; Mayer et al., 2011). Clinical outcomes may range from memory, attention, learning, and executive deficits (Strangman et al., 2012). The intrinsic interconnections between large-scale cerebral networks are essential for high-level cognitive functions, e.g., memory or attention. Post-TBI memory and attention impairments are correlated with abnormalities of the DMN and SN (Sharp et al., 2014). Salience is a major attentional mechanism which facilitates learning. Hypotheses may be made about which network is affected post-TBI (Barbey et al., 2015) and network diagnostics will certainly provide a powerful tool to refine the diagnosis-prognosis of occult mild TBI lesions.

*Fractal dimensions*, at the sub-level of intra-cellular scale via *micro-networks* (Foster, 2015) or *interactome-networks* control the physiological mechanisms of the selection process in determining the preferential attachment of synapses and affect by feedback the cartography of brain *macro-networks* (Foster, 2015). Hierarchical or fractal modularity of network topology exists for reconfiguration of connections between nodes (Bullmore and Sporns, 2012). This hierarchical modularity and *macro-networks* homeostasis rely on the optimal functionality of an intact genomic and proteomic intra-cellular system within *micro-networks*. In contrast, the opposite is not true and optimal operation of *macro-networks* does not seem necessary for proper *micro-networks* functionality. However, a hierarchy underlying the [*macro-networks*—*micro-networks*] relationship is claimed, with a sovereignty of the *macro-networks* over *micro-networks* (Foster, 2015). In the synaptic *macro-network*, the consolidation by repetitions of propagation of action potential (e.g., training), on the aging—fitness of nodes (synapses) seems instrumental to increase the synaptic density. In contrast, a disruption of the macro-network (e.g., ICNs) damages the structure of the network and prevents the normal brain plasticity and the potential of neurons to change their synaptic connections (Ashford and Jarvik, 1985). The lengthening of axons, sprouting of collateral ramifications, and remodeling allowing the dwelling of new synapses, and new cognitive and behavioral operations may be precluded by non-reversible pathological lesions as described in the next paragraph.

Upon directly breaking down the [*macro-network* - *micro-network*] system, mild TBI, by prompting focal damage or diffuse axonal injury (DAI; Andriessen et al., 2010), intertwined with neuroinflammation, further altering focal axolemma permeability, axonal swelling/transport (Andriessen et al., 2010) is disrupting axonal connections which follow the blockage of axonal transport, later inducing Wallerian degeneration. At this stage, the breakdown of the myelin sheath and axon cylinder are present (Andriessen et al., 2010). In parallel, neuroinflammation also promotes the spread of misfolded proteins (Heneka et al., 2014) and neurodegeneration associated with amyloid-β plaques and neurofibrillary tangles (Fotuhi et al., 2012; Sharp et al., 2014), characteristic of Alzheimer's disease. A terminal recovery may or may not follow; the quality of synaptic reorganization may be inconstant or ultimately abnormal. Disconnection of brain networks (Sharp et al., 2014) may be irreversible and further induce neurological or cognitive impairment such as relational memory described in Monti's paper.

Because irreversible post-TBI damage may occur and determination of longitudinal changes in connectomics is problematic (Goh et al., 2015), innovative diagnostic tools such as connectomic imaging, specifically for inter-regional connectivity (Irimia et al., 2014), are desired. Influenced by graph theory, the mapping of connectivity or connectogram is commonly pictured circularly by function-structural parceling and connections using a color code which enable showing missing or altered connections in patients (Irimia et al., 2012a). Integrity of white matter tracts is observed by diffusion tensor imaging (DTI) and further insights into ischemic penumbra and edematous regions perfused by cerebrospinal fluid are provided by fluid-attenuated-inversion recovery (FLAIR; Irimia et al., 2014). In a lower-scale fractal dimension, *micro-networks*, exhibiting similar patterns as *macro-networks* (Foster, 2015), may also be disrupted. Wrong assignments and characteristics specific to the micro-scale such as biophysical, biochemical and functional interactions of DNA (gene sequences), RNA or metabolites may be completely dysregulated and, therefore, biomarkers may be produced by this abnormal functioning and further serve for diagnotics purposes. Large amounts of biochemical-genetic and neuroimaging data may be submitted to high-throughput analysis (Dinov et al., 2014); the multi-center ENIGMA (“Enhancing Neuroimaging Genetics through Meta-Analysis”) Consortium is conducting a genome-wide association looking at single nucleotide polymorphisms (SNPs) and creating a database of neuroimaging and genetic data (Bis et al., 2012; Stein et al., 2012; Thompson et al., 2014; Hibar et al., 2015a,b). Genes modifying the hippocampal phenotype (e.g., *BDNF*), possibly upregulated by epigenetic factors such as skeletal muscle exercise, are known for synaptic molding and hippocampal plasticity and recent studies have shown that the Val66Met *BDNF* polymorphism has been involved in greater resilience and recovery potential of higher-order executive functions to TBI than Val66Val *BDNF* carriers (Barbey et al., 2014).

Ultimately, cognitive performance in normal aging proceeds along a continuum of slight and slow but progressive impairment as illustrated in the figure (Foster et al., 2011) which may be superimposed and augmented by the effects any TBI-induced delayed alteration of cognitive functions. A threshold is attained when the concurrent addition or succession of DAI, amyloid-β plaques and dysfunction of large-scale neural networks lead to clinical symptoms of mild cognitive impairment. Surprisingly, the decline in memory performance may not be proportional to the fraction of cells remaining in the hippocampus, which would result in a fair performance although significant neurodegeneration is already present (Foster et al., 2011). Indeed, in patients with mild TBI early in life, Monti et al. clearly observed a marked reduction of neural activity for relational memory processing later in life. However, some questions remain: to what extent do the observed lesions relate to decline in memory performance? If the decline is not proportional to the neural damage or network disconnection what would be the singularity of this relation? Is it only slightly accruing (Figure 1, Curve 2) the progressive continuum of normal aging (Figure 1, Curve 1)? Or, is there an acute discontinuity or inflection apart (Figure 1, Curve 3) from the progressive normal cognitive decline?

**Figure 1:**
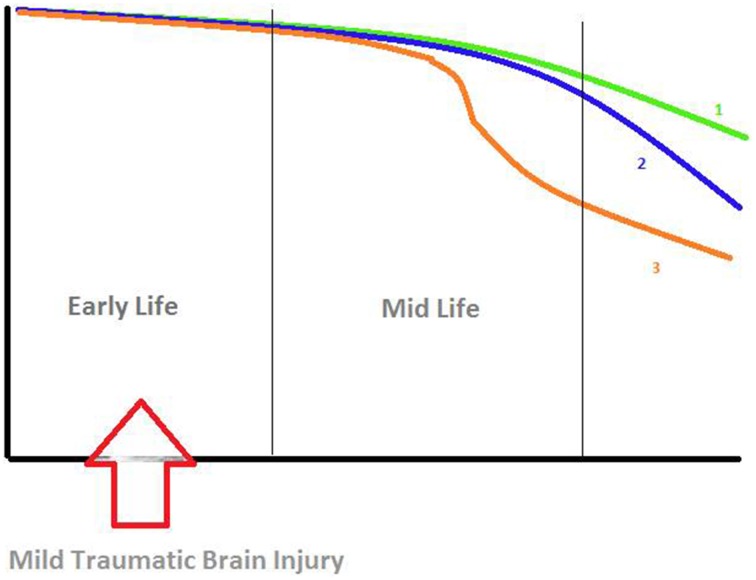
**Hypotheses about the decline in relational memory processing**. *X-Axis*: time (years), *Y-Axis*: memory performance. *Curve 1 (green)*: progressive continuum of normal aging. *Curve 2 (blue)*: Slight accrual of the progressive continuum of normal aging. *Curve 3 (orange)*: Acute discontinuity or inflection apart from the progressive normal cognitive decline. Figure derived from findings by various authors (Small et al., 2008; Foster et al., 2011; Smith et al., 2013).

## Conflict of interest statement

The author declares that the research was conducted in the absence of any commercial or financial relationships that could be construed as a potential conflict of interest.
